# Coordination strategies to improve COVID-19 PCR laboratory testing scale up in Nepal: An analysis

**DOI:** 10.1371/journal.pone.0314746

**Published:** 2024-12-05

**Authors:** Hannah Bakker, Arunkumar Govindakarnavar, Parvathy Krishnan Krishnakumari, Joaquim Gromicho, Fannie L. Côté, Nadia Lahrichi, Priya Jha, Saugat Shrestha, Rashmi Mulmi, Nirajan Bhusal, Deepesh Stapith, Runa Jha, Lilee Shrestha, Dhamari Naidoo, Reuben Samuel, Victor del Rio Vilas

**Affiliations:** 1 Health Care Lab, Karlsruhe Institute of Technology, Karlsruhe, Germany; 2 World Health Organization Country Office for Nepal, Kathmandu, Nepal; 3 Analytics for a Better World, Amsterdam, The Netherlands; 4 University of Amsterdam & ORTEC, Zoetermeer, The Netherlands; 5 CIRRELT & Polytechnique Montréal, Montréal, Quebec, Canada; 6 National Public Health Laboratory, Kathmandu, Nepal; 7 World Health Organization South East Asia Regional Office (WHOSEARO), New Delhi, India; 8 UK-Public Health Rapid Support Team, UK Health Security Agency, London, United Kingdom; Shree Nidhi Hospital Pvt. Ltd., NEPAL

## Abstract

During the COVID-19 pandemic, Nepal rapidly expanded its PCR testing capacity, essential for effective outbreak response. However, many laboratories faced overwhelming test volumes, resulting in delays that may have hindered containment efforts. This study aims to determine whether these challenges stemmed from capacity limitations and/or imbalanced sample distribution due to inadequate coordination. In this retro-perspective simulation of SARS-CoV-2 testing in Nepal during 2021, we evaluate the impact of coordinated sample transfers on reducing laboratory stress and wait times during demand peaks. Our findings reveal that centralized coordination and strategic partnerships for sample transfers significantly enhance diagnostic network performance, even under high demand. These insights offer valuable guidance for policymakers on implementing effective coordination strategies to strengthen diagnostic networks for future pandemics.

## Introduction

Diagnostic testing is a crucial component of effective outbreak response. It received significant attention in worldwide efforts to contain the spread of SARS-CoV-2 during the COVID-19 pandemic [1]. The PCR-based test to detect SARS-CoV-2 requires trained personnel, strict safety procedures, and equipment only found at specialized diagnostic laboratories. The sudden increase in demand for this test led to the quick expansion of the diagnostic network in several countries, including Nepal, the focus point of this study. Several studies evaluate the effectiveness of these scale-ups and report lessons learned. A common observation was the severe geographical imbalance of testing capacities. Furthermore, a lack of trained personnel and equipment and infrastructural challenges in resource-constrained areas led to immense constraints in the expansion of testing capacities, e.g., in Indonesia [2], Ghana [3], and Ethiopia [4]. Several studies suggest partnerships with private laboratories as a potential opportunity to increase capacities further [5, 6], yet, how these partnerships can be implemented remains an open question.

In Nepal, an increasing number of government-run laboratories were established and equipped with the necessary equipment to perform PCR testing. At the same time, several private laboratories started operating as high demand for diagnostic testing became a business opportunity. As of November 2021, 1.5 years into the pandemic, the diagnostic network in Nepal possessed significant capacity to perform PCR-based COVID-19 testing. Yet, particularly during temporally and geographically confined disease peaks, individual laboratories faced overwhelming sample numbers, resulting in overworked staff and long waiting times for test results. The following questions remain unaddressed up to this point:

1. Was the over-utilization of certain laboratories during high pandemic activity due to insufficient capacity, or did it result from an imbalanced distribution of samples caused by inadequate coordination?
2. What coordination strategies can effectively manage sample transfers during peak periods?

Nepal’s diagnostic network spans 7 administrative provinces, each varying in population, wealth, and access to laboratories. Most private laboratories have been established in the capital, Kathmandu, in Bagmati province. As each jurisdiction has its own funding, cross-province collaboration requires substantial administrative efforts; this limits sample transfers between provinces even if this option provides the optimal solution. Similarly, due to different finance models, collaborations between private and public laboratories posed difficulties. Given these challenges, this study also assesses whether cross-provincial collaboration between laboratories and public-private partnerships would result in improved test times.

Centralized coordination of test samples presents challenges, including the need for consistent data reporting, the organization of ad-hoc transfers, and the implementation of efficient transfer strategies. In consequence, coordination mechanisms have not been implemented throughout the pandemic. While the literature on the efficient design of diagnostic networks focuses on coordinating systems in a steady state, investigating strategies to efficiently respond to disease outbreaks and improve the system’s performance in times of distress has rarely been explored. For example, Jónasson et al. [7] reported diagnostic supply chains for early infant HIV detection in Sub-Saharan Africa. To the best of our knowledge, the only work focusing on the in-time coordination of samples is presented by Bakker et al. [8]. The authors present a rolling horizon procedure in which they determine near-optimal sample transfers in each period solely based on currently available data. We use this model to obtain a benchmark for the potential improvement attainable with sample transfer mechanisms. However, the focus of the present study lies in the identification of simple, implementable coordination strategies and the identification of focus points for fostering collaboration between stakeholders in pandemic/epidemic scenarios.

We are interested in coordination strategies that allow reducing the over-utilization of individual laboratories during periods of distress. Using data from the Delta (B.1.617.2) wave of the SARS-CoV-2 pandemic, this study explores the effectiveness of various coordination strategies through a retro-perspective simulation to inform scenarios of improved network performance, and simulates the impact of varying levels of collaboration on the performance and resilience of Nepal’s diagnostic network. The findings provide valuable insights into optimizing diagnostic network utilization and guiding future actions.

## Materials: SARS-CoV-2 Nepal 2021

The data used in this study comes from daily situation reports published by Nepal’s Ministry of Health and Population (MoHP) between May 1st and November 20th, 2021, covering various stages of the pandemic. These reports include the number of processed samples and positive test results from individual laboratories. Due to observed fluctuations in the reported numbers, which health officials attribute to delays in reporting rather than actual changes in test sample volumes, we use 7-day moving averages to smooth out these inconsistencies in our analysis.

We begin with a descriptive overview of the test sample distribution to motivate the research questions. Given that Bagmati, the most densely populated province and home to the capital, Kathmandu, processes significantly more test samples than any other province, we report results for each province individually. This approach prevents the situation in Bagmati from overshadowing national-level observations.

**Fig 1:**
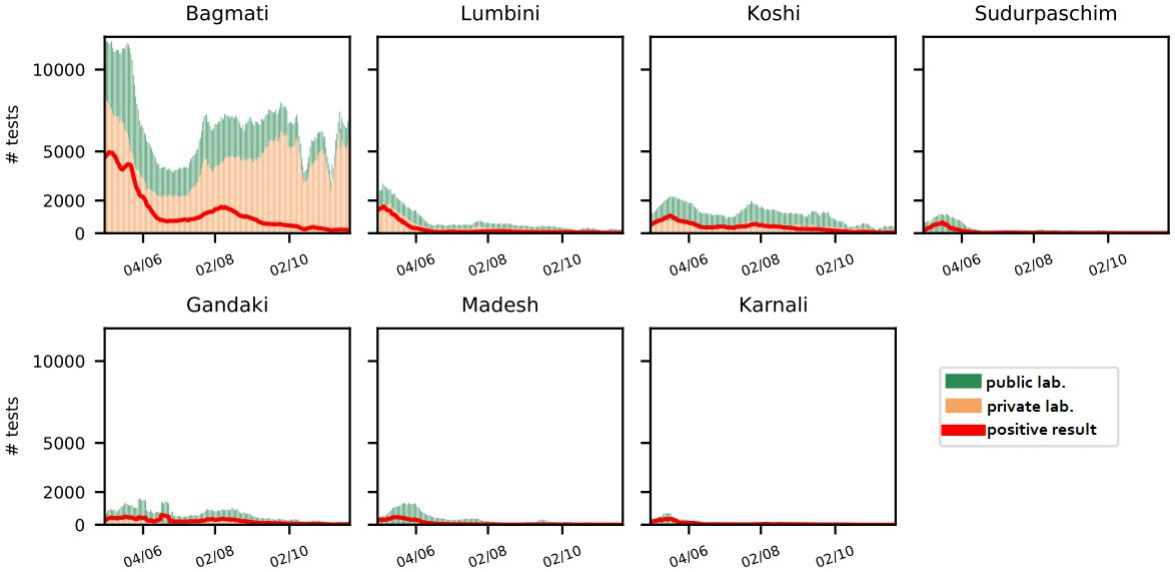
Testing activity per province. 7-day moving average of reported test sample numbers and positivity rates per province.

Fig 1 displays the number of test samples reported between May 1st and November 20th, 2021, for each province discriminating between public and private laboratories. The highest test volumes in each province occurred during the Delta (B.1.617.2) wave of the SARS-CoV-2 pandemic hitting Nepal in May and June 2021. Following this peak, the number of performed tests in Sudurpaschim, Madesh, and Karnali diminishes.

Private laboratories operate only in Bagmati, Lumbini, Koshi, and Gandaki, which together account for 65% of Nepal’s population [9]. Table 1 displays the total test volumes and the respective shares of tests performed by private and public laboratories. The share of private tests increases significantly from 47.2% in May to 72.5% in November. This shift is due to a steeper decline in the number of tests processed by public laboratories compared to private ones. Public laboratories reported a total of approx. 271K tests in May, reducing to 60K tests in October–a 77.9% decrease. In contrast, private laboratories report approx. 243K tests in May and only reduce to approx. 158K in October, a smaller decrease of 35.0%. Table 2 details the change in the share of public versus private tests by province over different time periods. The decline in public testing is mainly due to the testing activity in Bagmati, where the share of public tests decreased from 42.8% to 23.8%. This aligns with a change in the national testing strategy, which introduced charges for voluntary testing at public laboratories, matching the fees of private laboratories. This led to an increase in voluntary testing at private laboratories and a corresponding decrease at public facilities.

**Table 1:** Sample volumes per month for public and private laboratories at a national level.

Month	Total	Public	Private
**May**	513516	270912 (52.8%)	242604 (47.2%)
**June**	272982	166358 (60.9%)	10663 (39.1%)
**July**	256639	130507 (50.9%)	126131 (49.2%)
**August**	309891	138320 (44.6%)	171570 (55.4%)
**September**	287089	104349 (36.4%)	182740 (63.7%)
**October**	217428	59826 (27.6%)	157601 (72.5%)

**Table 2:** Fraction of sample volumes for different phases per province reported by government and private laboratories (public % / private %).

	Bagmati	Gandaki	Karnali	Lumbini	Koshi	Madesh	Sudurpaschim
**May-Jun**	42.8 / 57.2	79.3 / 20.7	100.0 / -	43.3 / 56.7	65.4 / 34.6	100.0 / -	100.0 / -
**Jul-Aug**	37.1 / 62.9	70.1 / 29.9	100.0 / -	46.6 / 53.4	64.3 / 35.7	100.0 / -	100.0 / -
**Sep-Oct**	23.8 / 76.2	63.6 / 36.4	100.0 / -	48.8 / 52.0	60.5 / 39.5	100.0 / -	100.0 / -

**Fig 2:**
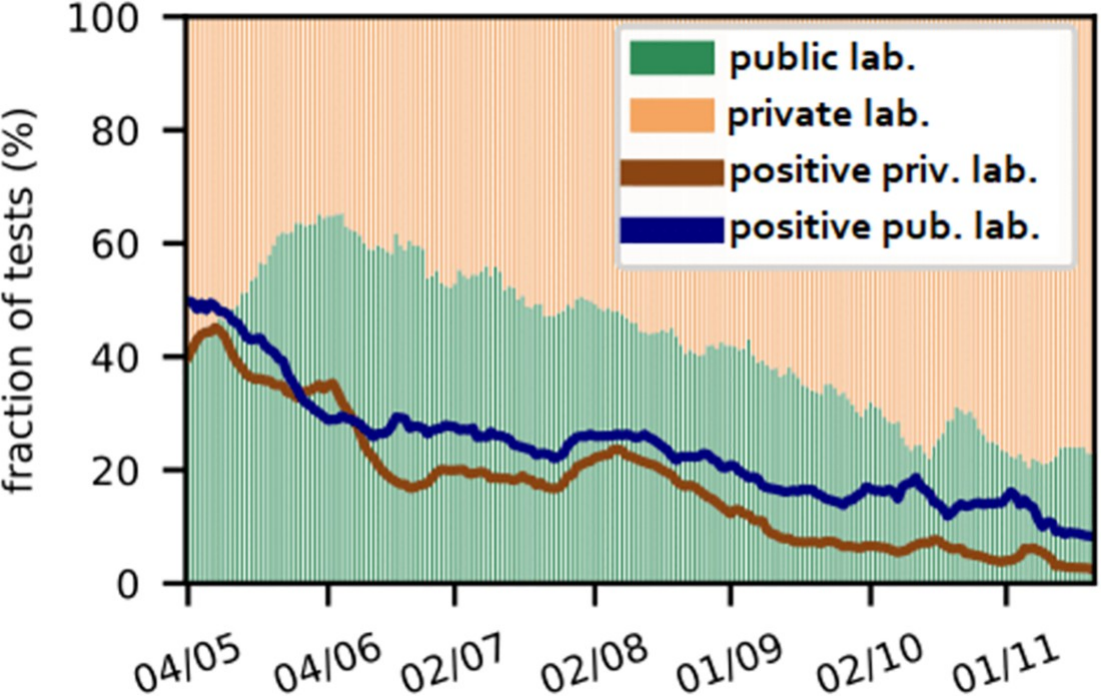
Testing activity at a national level. Share total number of tests performed by public and private laboratories as well as respective test positivity rates.

Fig 2 displays the test positivity rates (TPR) for public and private laboratories at a national level. The TPR at public laboratories is, on average, 6.27% higher than at private laboratories. The maximum difference of 12.14% in TPR was observed on November 2nd, 2021. This can be explained by the fact that public laboratories process all test samples from official case identification, contact tracing, and surveillance efforts and, therefore, they tend to receive samples more likely to yield a positive test result. In contrast, tests at private laboratories are more likely purchased by individuals for traveling or an earlier release from quarantine, thus less likely purchased by a symptomatic patient and more likely to be negative. Additionally, private laboratories account for the predominant part of the active testing, i.e., by mobile sample collection facilities actively moving into communities. The latter two points contribute to the fact that test volumes at private laboratories are less affected by the overall pandemic situation, as observed in Fig 2. The data shows that to get a comprehensive picture of the effectiveness of different coordination strategies in Nepal one must take the distinction between individual provinces and public and private laboratories into account.

**Fig 3:**
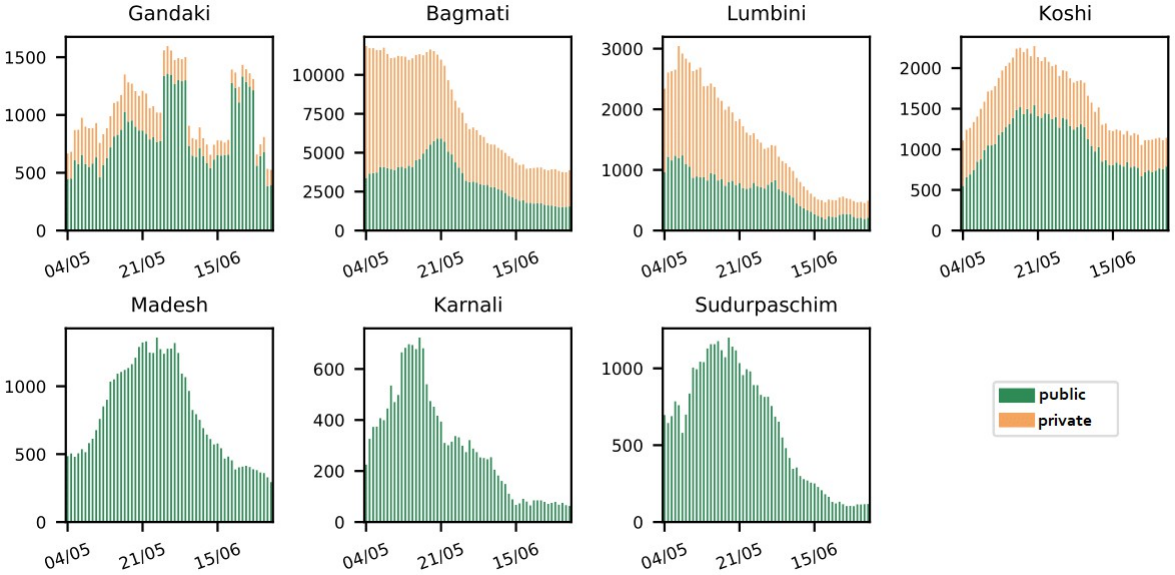
Sample volumes in baseline scenario. 7-day moving average of reported test sample numbers per province during Delta (B.1.617.2) wave.

We define the planning horizon of our retro perspective baseline scenario from May 3rd to July 1st, 2021, the time period of the Delta (B.1.617.2) wave of SARS-CoV-2 in Nepal. Each of the 57 days represents one period. The development of the accumulated sample numbers over this planning horizon is depicted in Fig 3 for public and private laboratories, respectively.

**Fig 4:**
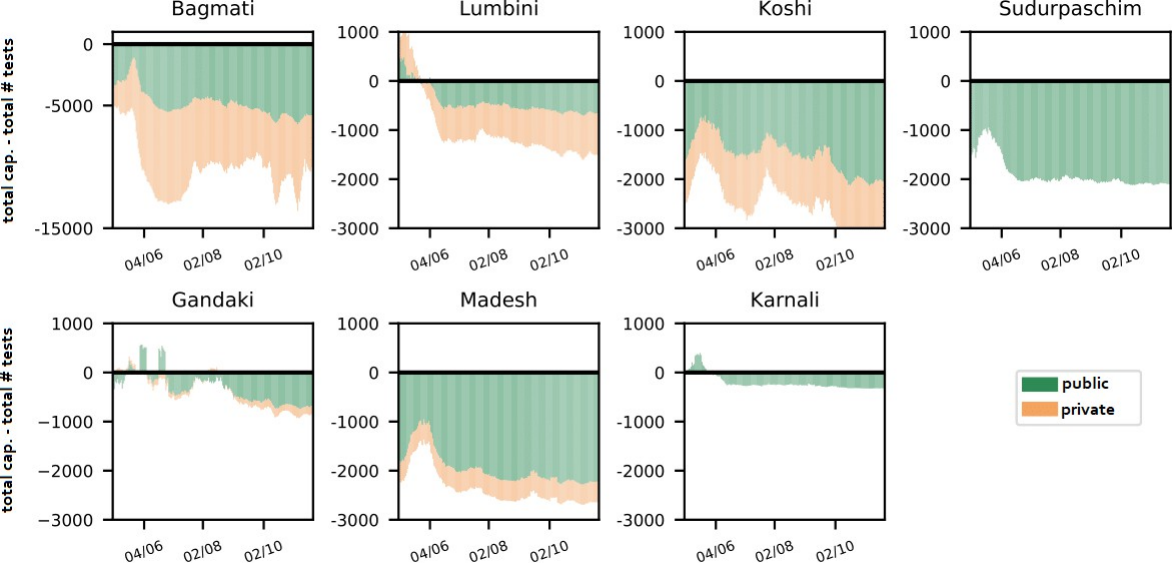
Estimated excess of diagnostic capacity. Accumulated capacity estimates minus daily throughputs per province.

Fig 4 depicts the overall capacity shortage or excess. It shows that the total testing capacity for most provinces consistently exceeded the total throughput for private and public laboratories. As a result, one may conclude that there is potential in a coordinated distribution of test samples. We will subsequently investigate the degree to which this holds when considering the geographical dispersion of demand and capacity within provinces.

## Methods

We use simulation to analyze the potential of coordinated sample transfers between laboratories to prevent over-utilization and, as a consequence, long waiting times for test results at individual laboratories during times of high pandemic activity. In the following, we describe our chosen performance measure, the fraction of delayed test samples, the simulation model, and the examined forms of coordination and collaboration.

### Performance measurement

The turn-around-time (TAT), the time between sample collection and result availability, is a central performance criterion for diagnostic networks [7]. This analysis only considers a subset of the processes or steps contributing to TAT, the laboratory waiting time and potential transfer travel time, so the TAT cannot be accurately measured. However, since waiting time directly impacts the TAT, reducing it shortens TAT. In this study, we define the target of 24 hours to start processing and consider a test sample “delayed” if transfer and waiting time together exceed this threshold. We assess our coordination mechanisms by measuring the fraction of delayed samples over the planning period [8].

### Simulation model

This simulation aims to retrospectively assess whether better coordination could have prevented overutilization in individual laboratories in 2021, focusing on whether the issue was an imbalance in sample distribution rather than a general lack of capacity. It is a discrete event simulation that simulates the arrival and sequential processing of test samples at laboratories [10]. Thereby, it monitors queues and expected waiting times at the laboratory entries. In Nepal, laboratories process SARS-CoV-2 samples on a first-in-first-out (FIFO) basis, meaning any excess samples in one period create a backlog, delaying samples in subsequent periods. S1 Fig illustrates this backlog process.

We assume the planning horizon is divided into periods (e.g., days), during which samples arrive at laboratories with known capacities to process samples in the queue. We estimate the waiting time of an incoming sample by comparing the current length of the queue to the laboratory’s processing capacity per period. If it exceeds one period, we consider transferring the sample to another laboratory within regional proximity. Transfers are determined by the coordination strategy and collaboration level, as explained next.

We set periods to workdays and define regional proximity by being accessible within an 8-hour drive, thus, a transfer possible between periods, e.g., overnight. Sample numbers come from daily situation reports, and travel times between laboratories are calculated using Google Maps. Due to unavailable data, laboratory processing capacities were estimated at 80% of the maximum reported throughput and validated by field experts.

### Coordination strategies

We test different coordination strategies in the simulation model. The first, *no coordination*, mimics the status quo in which each test sample is processed at the laboratory to which it is initially delivered. If incoming sample volumes exceed the processing capacities, the excess samples will be delayed. The results serve as a benchmark for the status quo.

To explore the potential of coordination to reduce overutilization, we assume an omniscient centralized authority that optimizes sample transfers in real-time, based on Bakker et al.’s near-optimal rolling-horizon framework [8]. We refer to this coordination strategy as *unrestricted coordination*. It provides a lower bound on the achievable fraction of delayed samples, indicating if overall diagnostic capacity is insufficient in a region.

Despite the efforts Nepalese health officials put into the persistent flow of data and information, achieving unrestricted real-time coordination would be challenging in practice due to communication and logistical constraints, and contribute additional burden on services. Therefore, we explore three simplified strategies:

1. Provincial Laboratory (*PL*): Each laboratory can only transfer samples to its designated provincial laboratory. All provincial laboratories are within an 8-hour drive from other public laboratories in their province, except in Sudurpaschim, where Kamalbazar Municipality PCR Lab cannot transfer samples due to a 10-hour drive to Seti Provincial Hospital. With this assignment strategy, Kamalbazar Municipality PCR Lab has no transfer possibility.
2. Cross-border provincial laboratory (*PL-cross province*): Laboratories can transfer samples to any provincial hospital within an 8-hour drive, even if it’s in a different province. This strategy allows all laboratories to transfer samples.
3. *n* Strategic Partner Laboratories (*n SP*): Each laboratory is paired with *n* strategic partners. Samples can only be sent to a partner laboratory if it has sufficient capacity to process them in the next period. These strategic partners are predetermined based on the laboratories that received the most transfers during the *unrestricted coordination* simulation.

### Collaboration levels

The coordination strategy determines how suitable transfers are identified, while the collaboration level dictates between which laboratories, within a feasible regional distance, these transfers are permitted. In Nepal, laboratories are categorized as public or private. Public laboratories are funded by their respective jurisdictions, making cross-provincial collaboration administratively challenging. Therefore, we distinguish between inner-province and cross-province collaboration, as well as public-only and public-private collaboration.

## Results

First, we model sample processing at individual laboratories without transfers (*no coordination*) to estimate the fraction of delayed samples, using this as a benchmark. Then, assuming a central authority coordinating real-time transfers using a near-optimal strategy (*unrestricted coordination*), we assess the potential benefits and suitable collaboration levels. Finally, we evaluate simplified coordination strategies and extend the experiments to test their validity across different scenarios.

### Potential of coordination and collaboration

Table 3 displays the fraction of delayed test samples for the period for both *no coordination* or *unrestricted coordination* for varying collaboration levels. Without coordination, 21.4% of samples missed the 24-hour processing target, with public laboratories experiencing a slightly higher delay rate (23.6%) than private laboratories (18.7%). Most delays occurred in rural provinces, including Gandaki, Karnali, and Sudurpaschim.

**Table 3:** Fraction of delayed samples total (public/private) per province for different levels of coordination and collaboration.

Coordination	No coordination	Unrestricted coordination (max. 8h)			
Collaboration		inner-province		cross-province	
		public-only	public-private	public-only	public-private
**Bagmati**	15.2 (16.7/14.1)	0.2	0 (0/0)	0	0 (0 / 0)
**Gandaki**	41.8 (38.2/55.4)	0	0 (0/0)	0	0 (0 / 0)
**Karnali**	30.8 (30.8/ -)	25	25 (25/-)	0	0 (0 / 0)
**Lumbini**	26.1 (16.4/33.5)	7.7	4.6 (3.2/5.6)	0	0 (0 / 0)
**Koshi**	22.9 (25.1/18.8)	0	0 (0/0)	0	0 (0 / 0)
**Madesh**	19.1 (19.1/-)	0	0 (0/-)	0	0 (0 / 0)
**Sudurpaschim**	47.7 (47.7/-)	28.4	28.4 (28.4/-)	0	0 (0 / 0)
**National**	**21.4 (23.6/18.7)**	**4.1**	**2.3 (3.6/0.8)**	**0**	**0 (0 / 0)**

With *unrestricted coordination* and appropriate collaboration, all samples can be processed on time in the simulation. Allowing to transfer samples only between public laboratories within the same province reduces national delay rate from 21.4% to 4.1%. The least improvement is seen in Karnali, Lumbini, and Sudurpaschim, which lacked private laboratories and had insufficient diagnostic capacity, as shown in Fig 4. Allowing transfers between public and private laboratories further reduces delays by 0.2% in Bagmati and 3.1% in Lumbini, but only lowers the national delay rate from 4.1% to 2.3% due to the absence of private laboratories in many rural provinces. Cross-provincial transfers to laboratories within an 8-hour drive eliminate all delays, mainly benefiting rural provinces like Karnali, Lumbini, and Sudurpaschim. These results highlight the significant potential of coordinated sample transfers, suggesting that cross-provincial collaboration between public laboratories should be prioritized, with collaboration with private laboratories as a secondary option.

Sample transfers require logistical effort. With cross-provincial collaboration among public laboratories, the *unrestricted coordination* strategy suggests 495 sample operations over 57 days, averaging 8.7 transfers per day. Notably, 80% of these transfers occur within the same province, while cross-province transfers are most significant in Gandaki (43.6%), Karnali (59.2%), and Lumbini (45.2%). These findings suggest that, with moderate logistical efforts, coordinated transfers can significantly reduce stress on individual laboratories during high pandemic activity, even in remote areas. The following analysis focuses exclusively on public-only cross-provincial transfers.

### Potential of simplified transfer strategies

**Fig 5:**
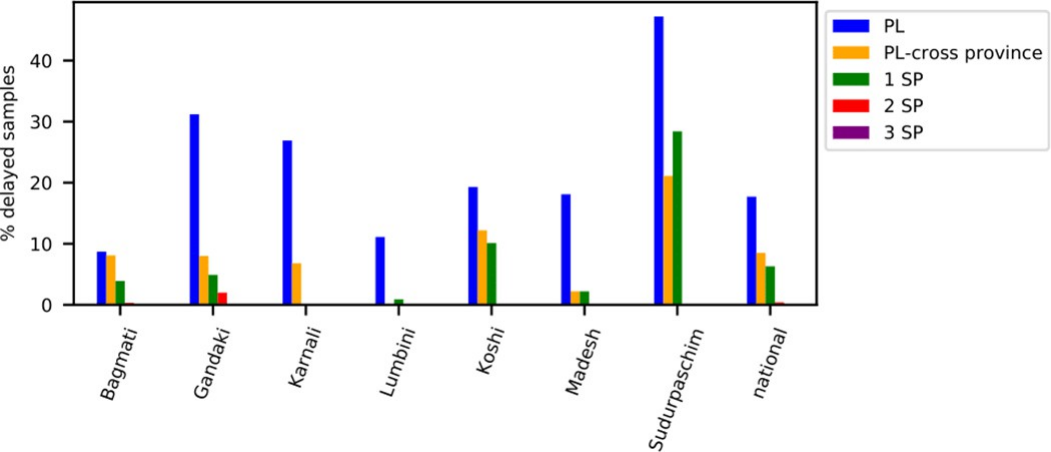
Performance of different coordination strategies in baseline scenario. Fraction of delayed samples with public-only cross-provincial collaboration for different coordination strategies.

Fig 5 displays the impact of different transfer strategies on the fraction of delayed samples with cross-provincial collaboration among public laboratories. Allowing transfers to provincial laboratories across provincial borders (*PL-cross province*) reduces the national delay rate from 21.4% to 8.5%. In contrast, restricting transfers to within the same province (*PL)* results in a national delay rate of 17.7%. Cross-provincial transfers significantly benefit rural provinces like Karnali, Lumbini, Sudurpaschim, and Gandaki. Comparing this strategy to using a set number of strategic partner laboratories (*n SP*) reveals that already assigning one strategic partner laboratory performs better than the *PL-cross province* strategy in all provinces except Sudurpaschim. Cooperation with two strategic partners reduces delays to 0.4%, and with three, delays are entirely eliminated.

In conclusion, enabling cooperation with one or two predefined strategic partner laboratories appears to be an effective strategy.

### Potential to increase resilience of the diagnostic network

Previous results were based solely on data from the second wave of the COVID-19 pandemic in 2021. We now simulate the proposed coordination and collaboration strategies under randomly generated scenarios with varying sample volumes and capacity estimates. This approach tests the robustness of our findings against errors in capacity estimation and assesses how coordination enhances the resilience of the diagnostic network to unforeseen changes. It also helps compare the value of increasing capacity versus implementing the proposed coordination strategies. The data from the second wave serves as a baseline scenario. We denote the incoming sample volumes at laboratory *i* in period *t* in the baseline scenario with $\bar{d_{it}}$ and its processing capacity with $\bar{Cap_{it}}$.

#### Increasing fluctuations of sample volumes

First, we introduce artificial fluctuations of a specified magnitude. The parameter Δ^*f*^ determines spread of these fluctuations, with larger Δ^*f*^ increasing the differences between the sample volumes arriving at two consecutive days. Thus, higher Δ^*f*^ raise the likelihood that a laboratory will experience significant spare capacity on one day and an overload the next. For each Δ^*f*^, we generate 30 scenarios in which the incoming sample volumes at each laboratory are drawn from the uniform distribution $U\left[\left(1-\Delta^{f}\right)\bar{d_{it}},\;\left(1+\Delta^{f}\right)\bar{d_{it}}\;\right]$. We vary Δ^*f*^ between 20% and 70%. Despite these fluctuations, the total demand from May 4th to July 1st remains consistent across scenarios, with a coefficient of variation of 0.6%.

**Fig 6:**
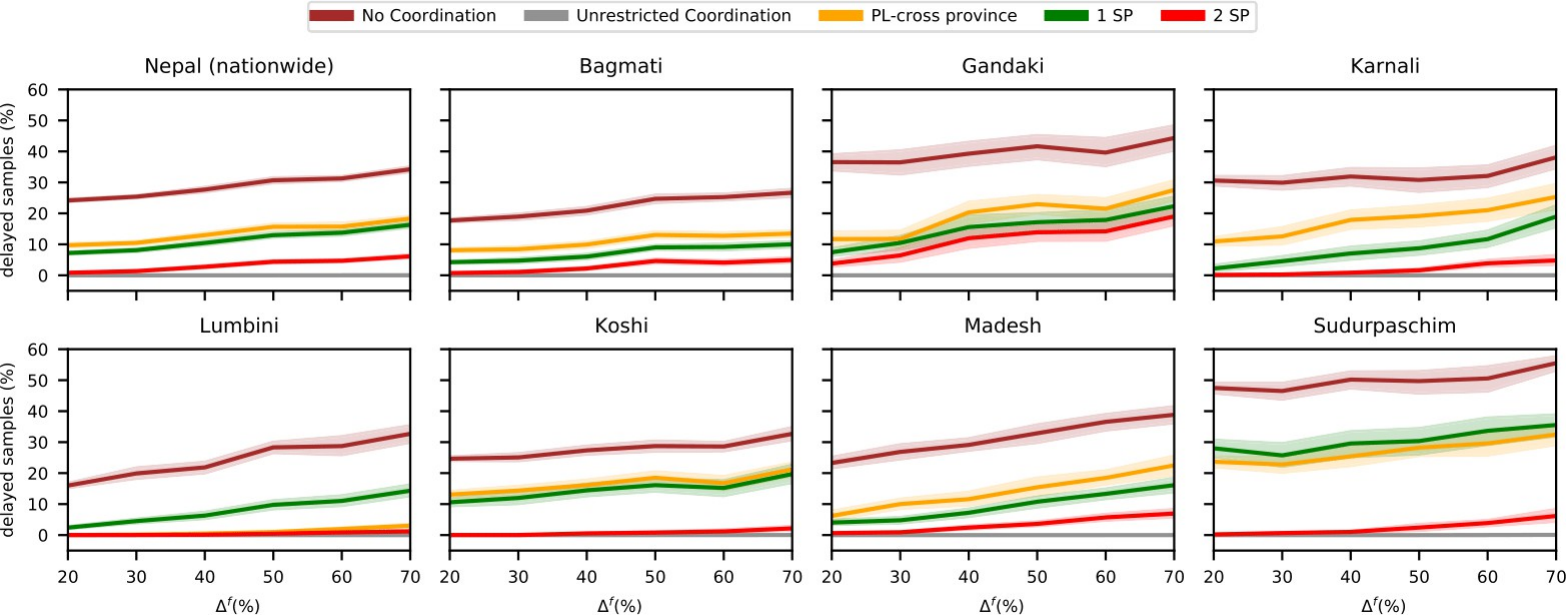
Performance of different coordination strategies for different levels of fluctuation of incoming samples. Fraction of delayed samples with public-only cross-provincial collaboration for different coordination strategies and different fluctuation levels of incoming samples.

Fig 6 depicts the average fraction of delayed samples and its 95% confidence interval for various coordination strategies at different fluctuation levels Δ^*f*^. As expected, increasing fluctuations in sample volumes lead to a higher fraction of delayed samples, as the likelihood of exceeding laboratory processing capacity rises. Without coordination, the national delay rate increases from 24.1% at Δ^*f*^ = 20% to 34.2% at Δ^*f*^ = 60%. Smaller provinces are more affected due to fewer laboratories, making individual performance more impactful.

In general, coordination proves effective in managing fluctuations. *Unrestricted coordination* eliminates delays across all provinces. Among simplified strategies, cooperation with two strategic partners consistently performs best, while cooperation with one strategic partner is the second-best option in all provinces except Lumbini. There, expanding the capacity of the provincial laboratory might be beneficial.

Table 4 reveals that flexibility in handling fluctuations requires increased logistical efforts. The number of transfers rises from an average of 510 to 664 as Δ^*f*^ increases from 20% to 60%, with the fraction of transferred samples more than doubling from 4.0% to 8.8%. Simplified coordination strategies reduce the total number of transfers. This suggests that avoiding high fluctuations in sample volumes is crucial. A practical implication of this is that, for active testing, it is advisable to distribute testing days more evenly throughout the week to mitigate these fluctuations.

**Table 4:** Number of transfer operations required for different strategies for different fluctuation levels (mean (standard deviation)).

	Δ^*f*^ = 20%			Δ^*f*^ = 60%		
	Unr. Coord.	PL-cr. Prov.	2 SP	Unr. Coord.	PL-cr. Prov.	2 SP
**Bagmati**	190.5 (12.7)	93.9 (6.3)	163.9 (9.2)	258.9 (19.5)	114.2 (12.1)	222.9 (12.1)
**Gandaki**	47.7 (6.0)	25.1 (2.9)	37.2 (4.8)	51.9 (10.7)	20.4 (4.6)	45.6 (9.3)
**Karnali**	25.4 (3.4)	10.2 (2.1)	18.8 (2.5)	25.7 (5.0)	12.5 (3.9)	22.0 (4.0)
**Lumbini**	46.1 (4.8)	40.8 (3.5)	50.7 (5.1)	65.0 (9.0)	48.5 (6.4)	68.2 (7.1)
**Koshi**	63.6 (5.1)	21.7 (4.5)	54.7 (4.2)	86.2 (11.8)	27.3 (5.7)	67.2 (5.6)
**Madesh**	82.4 (8.5)	35.2 (6.8)	72.1 (6.4)	115.0 (14.6)	40.7 (9.0)	99.0 (11.2)
**Sudurpaschim**	54.2 (4.5)	19.8 (2.2)	39.7 (3.5)	61.0 (8.1)	21.1 (4.3)	43.0 (5.5)
**Nationwide**	510.0 (19.4)	246.7 (15.7)	437.2 (12.9)	663.7 (12.9)	284.7 (29.6)	567.8 (20.4)

#### Increasing sample volumes

After increasing the fluctuations of test samples between consecutive days, we assess how rising sample volumes impact the diagnostic network. The parameter Δ^*i*^ denotes the relative increase in incoming sample volumes compared to the baseline scenario. For Δ^*i*^ ranging from 2.5% to 48.5%, we generate 30 scenarios in which incoming sample volumes are drawn from the uniform distribution $d_{it}∼U\left[0.8\left(1+\Delta^{i}\right)\bar{d_{it}},1.2\left(1+\Delta^{i}\right)\bar{d_{it}}\;\right]$. While the mean sample volume increases by Δ^*i*^, the daily sample volumes fluctuate by ∓20% around their mean in between different scenarios.

**Fig 7:**
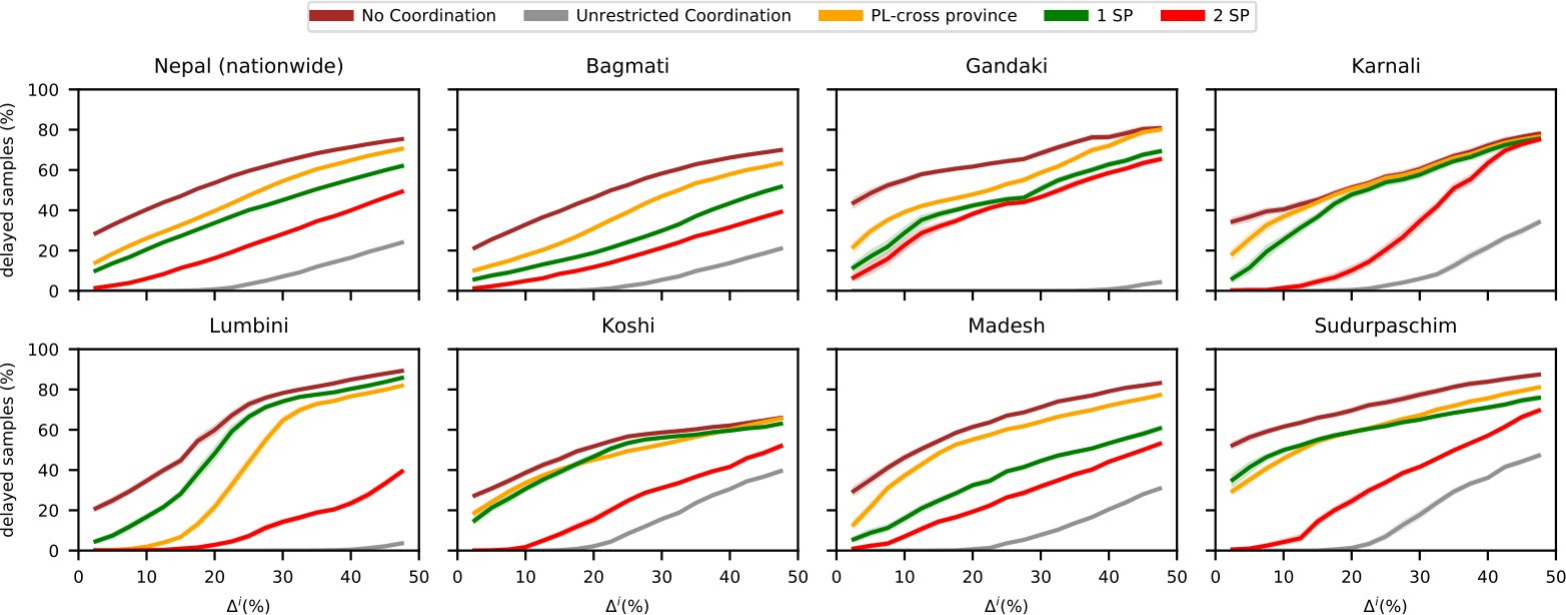
Performance of different coordination strategies for different increases of incoming samples. Fraction of delayed samples with public-only cross-provincial collaboration for different coordination strategies and different increases of incoming samples.

Fig 7 shows that higher sample volumes lead to more delayed samples across all provinces and strategies. The low delay rates under *unrestricted coordination* reveal the diagnostic network’s capacity to handle increased demand. Most provinces and the national level can process an average of 20% more samples without delays if transfers are efficiently managed. Without coordination, delays increase by approximately 25% with the same volume increase.

Gandaki or Lumbini can handle increases up to 40%, but in Lumbini, efficient coordination becomes crucial when sample volumes exceed a 20% increase. The performance difference between strategies *2 SP* and *PL-cross province* highlights this need. Simplified coordination strategies show that selecting two strategic partners significantly improves performance over one strategic partner. Cooperation with one strategic partner provides results similar to using the nearest provincial laboratory, both showing substantial improvement compared to *no coordination*.

#### Varying laboratory capacities

In the baseline scenario, maximum processing capacities were estimated at 80% of reported maximum throughput. To explore how varying these estimates affects strategy performance, we adjust capacity estimates uniformly across all laboratories. Let Δ^*c*^ denote the fraction of the maximum reported throughputs used as a capacity estimate for the individual laboratories, ranging between 65% and 95%. We generate 30 scenarios with sample volumes drawn from the uniform distribution $d_{it}∼U\left[0.8\bar{d_{it}},1.2\bar{d_{it}}\;\right]$.

**Fig 8:**
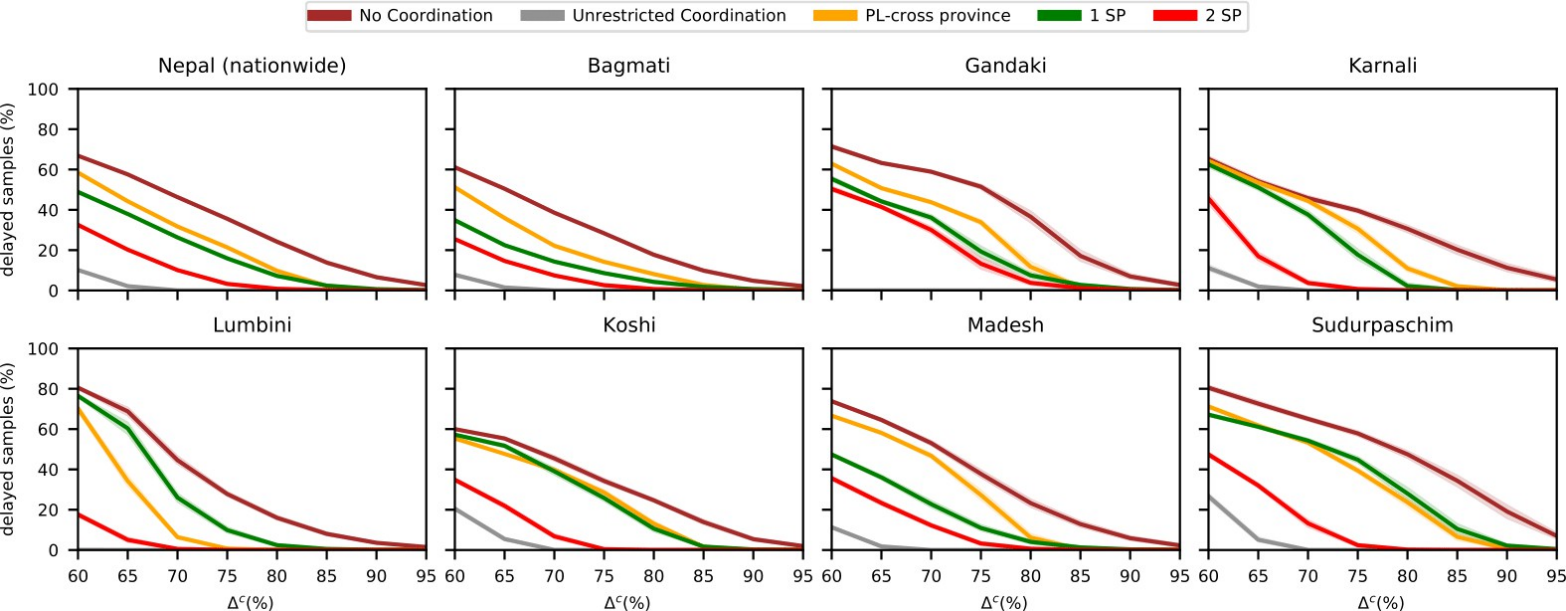
Performance of different coordination strategies for different capacity levels at individual laboratories. Fraction of delayed samples with public-only cross-provincial collaboration for different coordination strategies and different capacity levels at individual laboratories.

Fig 8 supports previous findings about the relative performance of coordination strategies. It shows that if effective coordination was in place, the overall capacities in the network could be reduced significantly while maintaining the service level. For example, at a capacity estimate of Δ^*c*^ = 80% (the same as the baseline), the average national delay rate across all scenarios is 24.2% when no coordination is possible. However, with transfers to two strategic partner laboratories, that rate drops to 0.9%. At a lower capacity level of Δ^*c*^ = 65% with the same strategy, the national delay rate averages 20.2%. Thus, by consistently applying the two partners strategy, we could reduce overall testing capacities by about 20% (1-65/80) while still outperforming the current system. This reduction would involve about 842 transfer operations on average (14.6 per day). In contrast, increasing current capacities by 15%, such that Δ^*c*^ = 95%, without enforcing any kind of coordination would still result in an average delay of 2.6%. Therefore, while coordination incurs additional logistical costs, it may be more effective than significantly increasing laboratory capacities to avoid the need for transfers.

#### Temporary capacity shortages

Lastly, we examine how temporary capacity shortages at individual laboratories impact results. Shortages, often due to issues like missing materials or staff illness, are defined by a shortage level Δ^*s*^ indicating the total capacity decrease per province. We generate 30 scenarios for each shortage level in which these shortages are randomly distributed across laboratories. If selected, a laboratory’s capacity reduces to a level between 25% and 75% of the original capacity for 1 to 7 periods. Laboratories, shortage volumes, and duration are randomly selected until the accumulated shortages equal the defined overall shortage volume.

**Fig 9:**
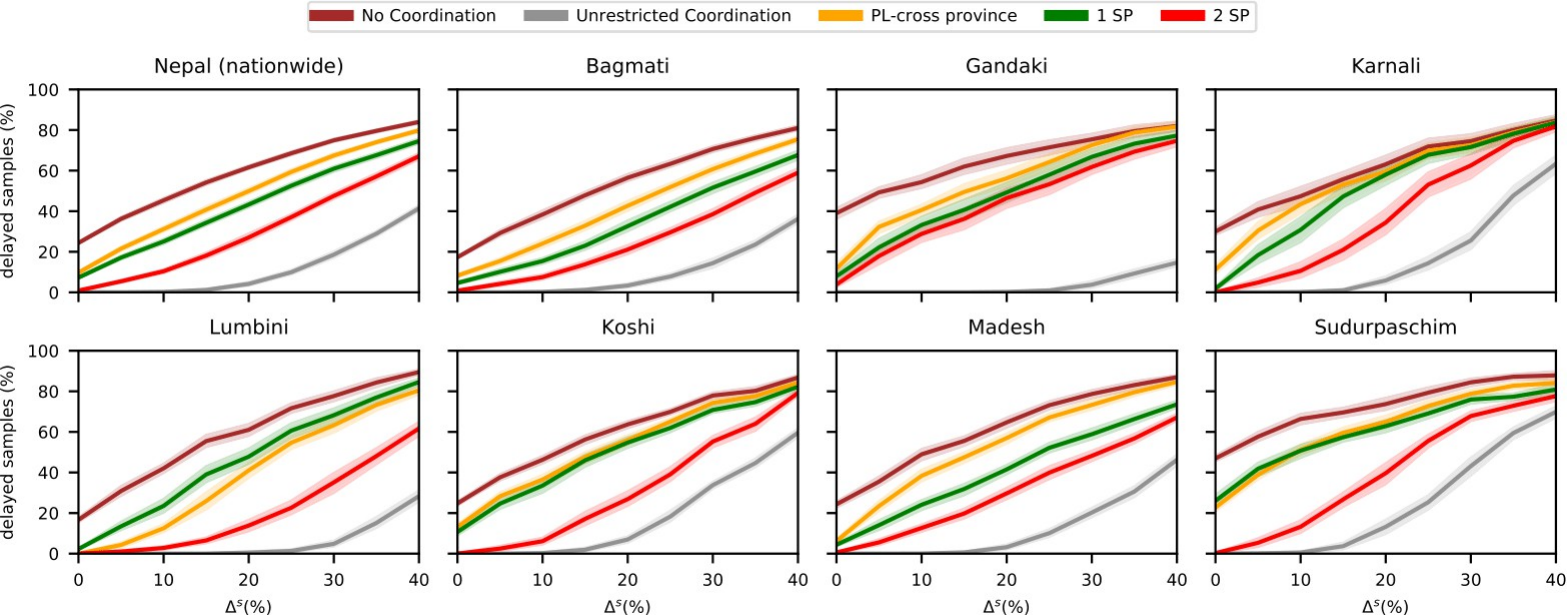
Performance of different coordination strategies for different levels of random capacity shortages. Fraction of delayed samples with public-only cross-provincial collaboration for different coordination strategies and different levels of random capacity shortages.

Fig 9 shows that increased shortage levels consistently lead to higher delays, with a more substantial impact compared to uniform capacity decreases. *Unrestricted coordination* manages to keep delays at a minimum, but delays begin to occur with approximately 20% random shortages across all provinces. At a national level, with Δ^*s*^ = 15% random shortages, the average delay rate is 1.2%. *Unrestricted coordination* significantly outperforms simplified coordination strategies, particularly in Gandaki, though simplified strategies still provide consistent improvements.

## Discussion

Our simulations demonstrate that coordinated sample transfers can significantly enhance the performance of Nepal’s diagnostic network. During the Delta wave from May 4th to July 1st, 2021, coordination could reduce the fraction of samples not processed within the 24-hour target from 21.4% to 0%. This suggests that Nepal’s overall processing capacity is adequate for high demand, but the issue of delays stems from geographically uneven demand peaks.

The diagnostic network in Nepal is composed of public laboratories in all 7 provinces and private laboratories in 4 of the 7 provinces, mostly in urban centers. Our results indicate that while collaboration between public and private laboratories is often advocated in the literature, its practical benefits in Nepal are limited. Private laboratories, located mainly in cities, cannot alleviate pressure in rural areas facing local outbreaks. They also face similar demand surges as public laboratories in cities, reducing their ability to support public testing efforts.

Our study also explored the effectiveness of various coordination strategies. An omniscient authority that initiates real-time sample transfers consistently outperformed simplified strategies. However, simpler strategies, such as those involving communication between one or two laboratories, also significantly reduced delays. Focusing on a few strategic partners for coordination proved more effective than constant transfers to provincial laboratories and should be prioritized when implementing coordination strategies.

Regarding resilience, coordinated sample transfers, even with basic strategies like transferring samples to a single predetermined partner, improved the diagnostic network’s robustness against capacity reductions and random shortages. In many cases, these strategies proved more effective than increasing individual laboratory capacities.

This study’s model is simplified and does not account for operational details such as sample arrival and transfer processes. It also assumes accurate data on the capacity and sample volumes available, which can be challenging to obtain in real time. Future research should integrate these operational aspects and account for uncertainties. Combining our simulation with a discrete-event simulation of the inner-laboratory processes could provide deeper insights. Despite these limitations, this study is pioneering in examining centralized sample coordination mechanisms and offers valuable directions for future research and policy development.

## Supporting information

S1 File Example on backlog generation during FIFO processing. (DOCX)

S2 File Inclusivity in global research questionnaire. (DOCX)

S1 Fig Effect of backlog at laboratory entry queue. (TIF)
