# Supplementary material for: Coordination strategies to improve COVID-19 PCR laboratory testing scale up in Nepal: An analysis
Source: PLoS One. 2024 Dec 5;19(12):e0314746. doi: 10.1371/journal.pone.0314746 (PMC11620682; doi:10.1371/journal.pone.0314746)
Supplement: S1 File — (DOCX) [file pone.0314746.s001.docx]

# Example on backlog generation during FIFO processing.

The example depicted in Figure 1 shows how the FIFO policy contributes to an overload of samples in one period, causing delays in several subsequent periods. Consider the following setting: the planning horizon is divided into periods of fixed length, e.g., days or half-days. Test samples arrive at a laboratory throughout one period to be processed within the next period. Each laboratory has a fixed and known processing capacity per period. The figure shows one laboratory over the course of 5 periods with a fixed processing capacity of 100 samples per period. The incoming sample volumes throughout the 5 periods are 150, 100, 80, 90, and 80. The laboratory can fully process the resulting total of 500 samples within the upcoming 5 periods. However, without the ability to transfer samples to other laboratories, the FIFO policy, together with the high number of test samples arriving in the first period, leads to a mitigation of delays throughout the planning horizon. In the end, 33% of the incoming 500 samples could not be processed within the next period. Meanwhile, the figure illustrates that if those 50 samples that exceeded the capacity in period 1 were transferred to another laboratory, the number of delayed samples could be reduced to zero. Therefore, transferring only 10% of the test samples may decrease the waiting time for test results for 33% of the test samples. The example exhibits a central idea behind the subsequently studied coordination strategies. If one manages to consistently avoid backlog from building up by transferring samples away from laboratories that are under distress to laboratories with spare capacities, one may significantly reduce the total stress in the system with relatively few logistical operations.

**S1 Fig. Effect of backlog at laboratory entry queue.** Visualization of the potential of sample transfers to reduce testing delays.
