# Supplementary figures and images for: Coordination strategies to improve COVID-19 PCR laboratory testing scale up in Nepal: An analysis
Source: PLoS One. 2024 Dec 5;19(12):e0314746. doi: 10.1371/journal.pone.0314746 (PMC11620682; doi:10.1371/journal.pone.0314746)

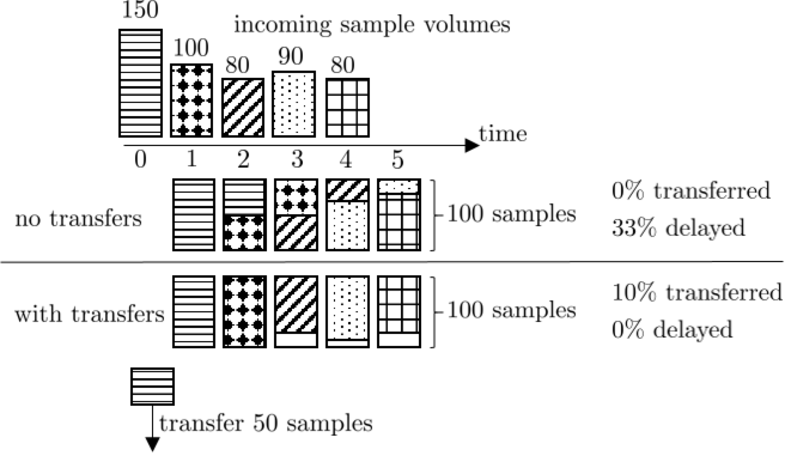

Supplement: S1 Fig — (TIF) [file pone.0314746.s003.tif]
